# High‐throughput Profiling of Pseudouridines in Microbiome‐derived Bacterial RNA

**DOI:** 10.1002/cpz1.70411

**Published:** 2026-07-01

**Authors:** Shikha Sharma, Ning Duan, Akintunde Emiola

**Affiliations:** ^1^ Microbial Therapeutics Unit, National Institute of Dental and Craniofacial Research National Institutes of Health Bethesda Maryland

**Keywords:** Gene regulation, microbiome, pseudouridine, post‐transcriptional modifications

## Abstract

Pseudouridine (Ψ) is a widespread RNA modification that influences RNA stability, structure, and translation. However, its role in bacterial mRNA, particularly within complex microbiomes, remains poorly defined. Here, we describe a bisulfite‐based sequencing workflow coupled with a scalable computational pipeline for base‐resolution, quantitative mapping of pseudouridine in microbiome transcriptomes. The protocol is optimized for low‐input, high‐complexity samples and includes strategies for efficient RNA extraction, ribosomal RNA depletion, bisulfite conversion, library preparation, and sequencing. The accompanying analysis pipeline enables detection and quantification of Ψ sites from chemically induced signatures, with modules for read alignment, site calling, and filtering in mixed‐bacterial datasets. This approach addresses key challenges in microbiome transcriptomics, including limited biomass, high rRNA content, and community heterogeneity. The protocol can be applied to samples from diverse microbial ecosystems to generate pseudouridylation profiles, enabling investigation of pseudouridine's role in post‐transcriptional regulation across microbial communities. Published 2026. This article is a U.S. Government work and is in the public domain in the USA. *Current Protocols* published by Wiley Periodicals LLC.

**Basic Protocol 1**: Microbiome sample collection and processing

**Basic Protocol 2**: mRNA enrichment and bisulfite treatment

**Basic Protocol 3**: cDNA synthesis and multiplexing of samples for sequencing

**Basic Protocol 4**: Computational pipeline for pseudouridine analysis

## Introduction

The human microbiome plays a central role in host physiology, influencing metabolic, immune, and signaling processes across organ systems (Gupta et al., 2022; Ma et al., 2024). Increasingly, microbiome research is shifting from descriptive, composition‐based analyses toward functional characterization using transcriptomics and other multi‐omics approaches (Jabs et al., 2020; Qiu et al., 2025; Wang et al., 2022; Xiao et al., 2022). These efforts aim to capture dynamic gene expression patterns that underlie microbial activity and host‐microbe interactions (Wang et al., 2025; Zhang et al., 2026). However, most microbiome transcriptomic studies focus on transcript abundance, with limited attention on post‐transcriptional regulatory mechanisms.

RNA modifications represent an important and underexplored layer of gene regulation within microbial communities (Chen et al., 2024; Wei & He, 2026). Among these, pseudouridine (Ψ) is one of the most abundant RNA modifications (Kim et al., 2010). Pseudouridine arises from the isomerization of uridine and enhances RNA stability, alters secondary structure, and influence translation efficiency and fidelity (Cerneckis et al., 2022; Kierzek et al., 2014). Although pseudouridylation has been extensively studied in eukaryotic systems (Martinez et al., 2022; Rodell et al., 2023) and in noncoding RNAs such as rRNA and tRNA (Yamagami et al., 2025; Zhao et al., 2023), its distribution and functional roles in bacterial mRNA, particularly within complex microbiomes, remain poorly characterized. This gap is particularly important because microbiome‐derived cell‐free RNA modifications may have the potential to serve as biomarkers for diseases such as colorectal cancer (Ju et al., 2026).

Transcriptome‐wide mapping of Ψ has been enabled by chemical approaches based on bisulfite‐mediated (BS) modification. In these methods, treatment of RNA with bisulfite reagents under controlled conditions generates adducts at Ψ residues that interfere with reverse transcription, producing characteristic sequence deletions in cDNA. By comparing BS‐treated and untreated samples, Ψ sites can be identified at single‐nucleotide resolution. Established protocols such as BID‐seq (bisulfite‐induced deletion sequencing; Dai et al., 2023) and PRAISE (pseudouridine assessment via sulfite/bisulfite treatment; Zhang et al., 2023) have demonstrated the effectiveness of this approach in eukaryotic systems. More recently, these methods have been extended to bacteria species (Xu et al., 2025). However, they are not directly applicable to microbiome‐derived RNA.

**Figure 1:**
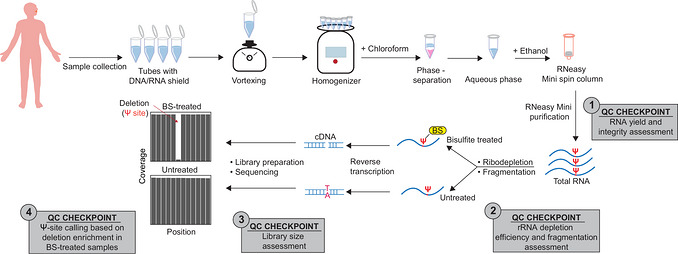
Workflow for pseudouridine profiling in microbiome samples. Schematic overview of experimental methods used in this protocol. Bisulfite treatment of RNA induces characteristic deletions at pseudouridine sites during cDNA synthesis. After sequencing, deletion signals in treated samples are compared with those of untreated controls to enable accurate identification of Ψ positions.

Microbiome‐derived RNA introduces several challenges. Complex microbial communities often contain substantial host RNA contamination, highly uneven species abundances, strain‐level sequence heterogeneity, and incomplete reference genome representation. These features can generate ambiguous alignments and background deletion signals that complicate accurate Ψ identification. To address these challenges, we recently developed a streamlined method for base‐resolution mapping of pseudouridine in microbiome transcriptomes (Sharma et al., 2026). The present workflow (Fig. 1) combines optimized RNA extraction from low‐biomass samples with efficient ribosomal RNA depletion to enrich for microbial mRNA (Basic Protocol 1). Controlled RNA fragmentation and bisulfite treatment conditions are used to generate Ψ‐dependent adducts while preserving RNA integrity (Basic Protocol 2). Reverse transcription and library preparation steps are adapted to capture modification‐induced signatures, and are followed by Illumina‐compatible sequencing (Basic Protocol 3). In addition, we provide a computational pipeline designed for microbiome transcriptomes. This pipeline incorporates strategies for read alignment, detection of Ψ‐associated sequence signatures, and filtering to reduce false positives arising from sequencing errors or mapping ambiguity in complex communities (Basic Protocol 4).

Together, this integrated experimental and computational framework enables high‐throughput mapping of pseudouridine landscapes across both defined microbial systems and complex microbiomes.

## Basic Protocol 1 MICROBIOME SAMPLE COLLECTION AND PROCESSING

Microbiome‐derived samples are typically characterized by low microbial biomass, substantial host nucleic acid contamination, and limited RNA yield, all of which complicate reliable detection of Ψ. Here, we present an optimized workflow that enables efficient recovery of high‐quality RNA from low‐biomass inputs, suitable for robust transcriptomic and RNA modification analyses. This workflow has been validated using oral subgingival plaque microbiome samples, demonstrating robust RNA yield and quality suitable for sequencing‐based Ψ profiling (Sharma et al., 2026).

### Materials

Bacterial RNA source of interest
DNA/RNA Shield Stabilization Solution (Zymo Research, R100‐50)
QIAzol Lysis Reagent (Qiagen, 79306)
Chloroform (Sigma Aldrich, 319988)
EMSURE Ethanol (Sigma Aldrich, 1.00983.1011)
RNeasy Mini kit (Qiagen, 74104)
DNA‐freeDNA Removal Kit (Thermo Fisher Scientific, AM1906)
DEPC‐treated nuclease‐free water (Thermo Fisher Scientific, AM9920)

Gracey curette (Hu‐Friedy Group)
Ice bucket
Vortex mixer (e.g., Sigma Aldrich, Z258423‐1EA)
2‐ml ZR BashingBead Lysis Tubes (0.1‐ and 0.5‐mm beads; Zymo Research, S6012‐50)
High‐speed homogenizer: e.g., MP BiomedicalsFastPrep‐24 Bead Beating Grinder and Lysis System (Fisher Scientific, 11‐600‐5500)
Benchtop centrifuge (e.g., Eppendorf, EP5405000646‐1EA)
Thermomixer (e.g., Eppendorf, T1317‐1EA)
10‐, 200‐, and 1000‐µl pipets and tips
1.5‐ml low‐binding tubes, sterilized (Thermo Fisher Scientific, 90410)

#### Sample collection and processing

1 Collect microbiome samples using sterile techniques and RNase‐free materials (e.g., in prior work, oral subgingival plaques were obtained using sterilized curettes into 1.5‐ml microcentrifuge tubes containing 65 µl of DNA/RNA stabilization reagent). Immediately place samples on ice or store at −80°C until further processing.
2 Thaw the samples on ice and vortex for 30‐60 sec to ensure complete resuspension in the stabilization buffer.
3 Transfer the entire sample to 2‐ml bead‐beating lysis tubes containing mixed‐size beads (0.1 mm and 0.5 mm).
4 Add 600 µl of a phenol‐based lysis reagent (e.g., QIAzol) to each tube to facilitate efficient disruption of microbial cells.
5 Perform sample lysis by bead‐beating using a high‐speed homogenizer for a total of 5 min at maximum speed. Perform homogenization in cycles of 1 min of homogenization followed by 5 min cooling on ice to minimize heat‐induced RNA degradation.
6 Centrifuge the homogenized samples for 2 min at ≥12,000 × *g*, room temperature, to pellet cellular debris. Carefully transfer the supernatant to fresh tubes.
7 Transfer ∼200 µl of the clarified homogenate to a new tube and add 180 µl chloroform. Mix thoroughly by inversion for 15‐20 sec and incubate at room temperature for 5 min. Centrifuge for 15 min at ≥12,000 × *g*, 4°C, to achieve phase separation.
8 After centrifugation, three phases will be visible: a clear upper aqueous phase (RNA), an interphase (DNA), and a lower organic phase (proteins). Carefully transfer the upper aqueous phase to a new tube without disturbing the interphase. Add 100% ethanol at a 1.5:1 (v/v) ethanol/aqueous phase ratio and mix thoroughly by pipetting.

#### Column‐based RNA purification

9 Load the mixture onto RNeasy Mini spin column (RNeasy Mini kit) placed in a 2‐ml collection tube. Centrifuge 20 sec at 8000 × *g*, room temperature, and discard the flowthrough.
10 Wash the spin column by adding 700 µl Buffer RWI (RNeasy Mini kit). Centrifuge for 20 sec at 8000 × *g*, room temperature, and discard the flowthrough. Add 500 µl Buffer RPE (RNeasy Mini kit) to the spin column. Centrifuge 2 min at 13,000 × *g*, room temperature, and discard the flowthrough and collection tube.
11 Place the RNeasy Mini spin column in a clean 1.5‐ml collection tube. Add 17 µl RNase‐free water directly to the center of the membrane. Incubate for 1 min and then centrifuge 2 min at 8000 × *g*, room temperature, to elute RNA.

#### Genomic DNA removal

12 To the purified RNA, add 0.1 vol of 10× DNase I buffer and 1 µl rDNase I (DNA‐free DNA Removal Kit). Mix gently by pipetting.
13 Incubate samples at 37°C for 20‐30 min in a thermomixer or heat block.
14 Add 0.1 volume of resuspended DNase Inactivation Reagent (DNA‐free DNA Removal Kit) to each sample and mix thoroughly by pipetting.
  If 0.1 volume for DNase Inactivation Reagent is <2 µl, add 2 µl of the reagent.
15 Incubate samples at room temperature for 2 min, mixing intermittently by gentle inversion.
16 Centrifuge samples 90 sec at 10,000 × *g*, room temperature, and carefully transfer the clear supernatant into a fresh tube. The supernatant contains DNase‐treated RNA suitable for downstream processing.

## Basic Protocol 2 mRNA ENRICHMENT AND BISULFITE TREATMENT

Total RNA isolated from microbiome samples is typically dominated by rRNA, resulting in poor representation of mRNA in sequencing libraries. To enrich for mRNA, rRNA depletion is performed using the Illumina Ribo‐Zero Plus rRNA Microbiome Depletion Kit. This method employs a targeted enzymatic strategy (ribodepletion) in which sequence‐specific capture probes hybridize to rRNA molecules and recruit RNase H for selective cleavage of RNA–DNA hybrids. Residual DNA probes are subsequently removed by DNase I, and then bead‐based purification is performed to recover the enriched RNA fraction (Wahl et al., 2022). Notably, this system is designed to efficiently deplete multiple rRNA species (5S, 16S, and 23S) across a broad spectrum of microbial taxa, including diverse host‐associated bacteria as well as host‐derived rRNA, thereby substantially improving the representation of microbiome mRNA in downstream analyses.

*IMPORTANT NOTE*: Efficient ribodepletion requires accurate RNA quantification and quality assessment (e.g., using a Qubit and/or Agilent Bioanalyzer). Samples should have a minimum RIN ≥5, with 25‐500 ng of total RNA in 11 µl input for optimal performance. All steps must be performed on ice, unless stated otherwise.

### Materials

RNA samples (Basic Protocol 1)
Qubit RNA High Sensitivity (HS) assay kit (Thermo Fischer Scientific, Q32852)
DEPC‐treated nuclease‐free water (Thermo Fisher Scientific, AM9920)
Agilent RNA 6000 Nano kit (Agilent Technologies, 5067‐1511)
Illumina Stranded Total RNA Prep, Ligation with Ribo‐Zero Plus Microbiome kit (Illumina, 20072063)
AMPure RNAClean XP beads (Beckman Coulter, A63987)
RNA Fragmentation Reagents (Thermo Fisher Scientific, AM8740)
RNA Clean and Concentrator‐5 column kit, (Zymo Research, R1015)
EMSURE ethanol (Sigma Aldrich, 1.00983.1011)
RNA Desulphonation buffer (Zymo Research, R5001‐3‐40)

Qubit fluorometer (Fisher Scientific, Q33327)
Agilent 2100 Bioanalyzer
Thermomixer (e.g., Eppendorf, T1317‐1EA)
Thermocycler (e.g., Fisher Scientific, A24811)
10‐, 200‐, and 1000‐µl pipets and tips
1.5‐ml low‐binding tubes, sterilized (Thermo Fisher Scientific, 90410)

#### mRNA enrichment

1 Quantify RNA samples using Qubit fluorometer and assess RNA integrity using a Bioanalyzer or equivalent system. Normalize each sample to a final volume of 22 µl with nuclease‐free water. Split the normalized RNA into two equal aliquots (11 µl each) and label as “treated” and “untreated” for downstream processing.
2 Deplete rRNA from both “treated” and “untreated” samples using the Illumina Stranded Total RNA Prep with Ligation (Ribo‐Zero Plus Microbiome kit) according to the manufacturer's instructions. After depletion, purify RNA using AMPure RNA XP beads according to the manufacturer's protocol.
  Ensure that AMPure RNA XP beads are equilibrated to room temperature for 30 min before use.

#### Fragmentation

3 Fragment RNA by adding 0.9 µl RNA Fragmentation Reagents to each sample and incubating at 95°C on a thermomixer for 20 sec.
4 Immediately add 0.9 µl of stop reagent (RNA Fragmentation Reagents) to terminate the reaction and place immediately on ice.
*CAUTION*: Avoid extended incubation to prevent over‐fragmentation or excessive RNA degradation.

#### Purification of untreated samples

Perform all steps in this section at room temperature.

5 Add 20 µl of binding buffer (RNA Clean and Concentrator‐5 kit) and 30 µl of 100% ethanol to each untreated sample. Mix thoroughly by pipetting.
6 Transfer the entire mixture (∼60 µl) to a Zymo‐Spin IC Column (RNA Clean and Concentrator‐5 kit) placed in a collection tube. Centrifuge 30 sec at 10,000 × *g*. Discard the flowthrough.
7 Add 400 µl of RNA Prep Buffer (RNA Clean and Concentrator‐5 kit) to the column and centrifuge 30 sec at 10,000 × *g*. Discard the flowthrough.
8 Add 400 µl of RNA Wash Buffer (RNA Clean and Concentrator‐5 kit) and centrifuge 1 min at 10,000 × *g*. Discard the flowthrough.
9 Transfer the column to a clean 1.5‐ml tube. Add 11 µl of DNase/RNase‐free water directly to the center of the column membrane. Incubate for 1 min and then centrifuge 1 min at 10,000 × *g*, to elute RNA. Immediately place samples on ice.

#### Bisulfite conversion of treated samples

Perform all steps in this section at room temperature.

10 Prepare fresh bisulfite reagent (BSR; see recipe in Reagents and Solutions) immediately before use and mix thoroughly until completely dissolved.
11 Add 45 µl of freshly prepared BSR to 11 µl fragmented “treated” RNA sample. Incubate at 70°C for 3 h in a thermocycler.
12 After incubation, add 75 µl nuclease‐free water to the reaction and mix. Add 270 µl RNA binding buffer (RNA Clean & Concentrator‐5 kit) and mix thoroughly. Then add 400 µl of 100% ethanol and mix thoroughly by pipetting.
13 Transfer the entire mixture (∼800 µl) to a Zymo‐Spin IC column (RNA Clean and Concentrator‐5 kit) placed in a collection tube. Centrifuge 30 sec at 10,000 × *g* and discard the flowthrough.
14 Add 200 µl RNA Wash Buffer (RNA Clean and Concentrator‐5 kit) to the column and centrifuge 30 sec at 10,000 × *g*. Discard the flowthrough. Add 200 µl RNA desulphonation buffer to the column and incubate for 75 min.
15 Centrifuge the column for 30 sec at 10,000 × *g* to remove the buffer. Discard the flowthrough. Add 400 µl of RNA Wash Buffer and centrifuge 1 min at 10,000 × *g*. Discard the flowthrough.
16 Transfer the column to a clean 1.5‐ml tube. Add 11 µl of DNase/RNase‐free water directly to the center of the membrane. Incubate for 1 min and then centrifuge 1 min at 10,000 × *g* to elute RNA. Immediately place samples on ice.

## Basic Protocol 3 cDNA SYNTHESIS AND MULTIPLEXING OF SAMPLES FOR SEQUENCING

Although the Illumina library preparation kit used in this protocol includes a double‐stranded cDNA synthesis module, its chemistry is not compatible with bisulfite‐treated RNA and should not be used for these samples. Instead, first‐strand cDNA synthesis is performed using SuperScript IV Reverse Transcriptase, which provides improved efficiency and fidelity when reverse‐transcribing chemically modified RNA (Zhang et al., 2024). Furthermore, efficient detection of Ψ‐induced deletion signatures depends on robust reverse transcription across bisulfite‐induced adducts; therefore, use of SuperScript IV Reverse Transcriptase is recommended for optimal sensitivity and reproducibility.

### Materials

Eluted fragmented and bisulfate‐treated, as well as untreated, RNA (Basic Protocol 2)
50 µM random hexamers (IDT)
Deoxynucleotide (dNTP) Solution Mix (NEB, N0447S)
DEPC‐treated nuclease‐free water (Thermo Fisher Scientific, AM9920)
SuperScript IV Reverse Transcriptase kit (Thermo Fisher Scientific, 18090010)
Dithiothreitol (DTT)
RNaseOUT Recombinant Ribonuclease Inhibitor (Thermo Fisher, 10777019)
Bovine serum albumin (BSA; Sigma Aldrich, A2153)
0 U/µl DNA polymerase I (Thermo Fisher, EP0041)
5 U/µl RNase H (Thermo Fisher, EN0202)
Ampure XP beads (Beckman Coulter, A63881)
Illumina DNA/RNA UD Indexes Set A (Illumina, 20091654)
Illumina Stranded Total RNA Prep with Ligation, Ribo‐Zero Plus Microbiome kit (Illumina, 20072063)

DNA 1000 kit for 2100 Bioanalyzer (Agilent)
Thermocycler (e.g., Fischer Scientific, A24811)
10‐, 200‐, and 1000‐µl pipets and tips
1.5‐ml low‐binding tubes, sterilized (Thermo Fisher Scientific, 90410)
Agilent 2100 Bioanalyzer

#### Annealing of random hexamers

1 To the eluted RNA, add 1 µl of 50 µM random hexamers, 1 µl of 10 mM dNTP mix, and 4 µl nuclease‐free water. Mix gently and incubate at 65°C for 5 min in a thermocycler, and then immediately place samples on ice.

#### First‐strand cDNA synthesis

2 Prepare a reverse transcription master mix in a microcentrifuge tube according to the table below. Scale the volumes proportionally based on the total number of samples, including excess volume to account for pipetting loss.

**Table:** 

Component	Volume per reaction
5× SuperScript IV buffer	4 µl
10 mM dNTP mix	1 µl
0.1 M DTT	1 µl
RNaseOUT RNase Inhibitor	1 µl
BSA (20 µg/µl)	0.4 µl
SuperScript IV Reverse Transcriptase (200 units/µl)	1 µl
3 Add 8 µl of the reverse transcription master mix (step 2) to each sample from step 1. Perform first‐strand cDNA synthesis using the following thermocycler program:
  10 min at 23°C
  60 min at 50°C
  10 min at 80°C
  Immediately place samples on ice.

#### Second‐strand cDNA synthesis

4 Prepare second‐strand synthesis reaction by combining the following components in a microcentrifuge tube:

**Table:** 

Component	Volume per reaction
DNA polymerase I reaction buffer	4 µl
DNA polymerase I	3 µl
RNase H	0.8 µl
Nuclease‐free water	12.2 µl
First‐strand cDNA (from step 3)	20 µl
  Incubate the reaction in a thermocycler using the following program:
  2 h at 15°C
  10 min at 75°C
5 Purify cDNA using AMPure XP beads according to the Illumina Stranded Total RNA Prep, Ligation with Ribo‐Zero Plus (Microbiome) Reference Guide (cDNA cleanup after second‐strand synthesis). Add 90 µl of AMPure beads to each sample and proceed according to the manufacturer's instructions (see reference guide, p. 15).

#### Multiplexing

6 Perform 3′ adenylation, adapter ligation, and indexing PCR for each sample using the Illumina Stranded Total RNA Prep, Ligation with Ribo‐Zero Plus (Microbiome) kit according to the manufacturer's instructions (see reference guide, p. 16). Assess size distribution and quality of the resulting library using an Agilent 2100 Bioanalyzer with a DNA 1000 kit. Proceed with high‐throughput sequencing using an Illumina platform according to standard protocols.

## Basic Protocol 4 COMPUTATIONAL PIPELINE FOR PSEUDOURIDINE ANALYSIS

Analysis of microbiome transcriptomes for Ψ detection requires a tailored computational workflow to account for mixed species composition. The first step is the construction of an appropriate reference genome database for read alignment. Because comprehensive microbial genome databases can substantially increase computational load and runtime, it is strongly advisable to restrict the reference set to taxa likely to be present in the samples. Where prior sequencing data or taxonomic profiles are available, the reference database can be further refined based on coverage. For example, inclusion of genomes with ≥20 × coverage in both bisulfite‐treated and untreated samples can enhance computational efficiency in complex microbiome datasets. The pipeline is located at https://github.com/EmiolaLab/bacPseudouridines.

### Hardware

PC with access to high‐performance computing (HPC)

#### Software

BWA (tested on v0.7.17)
SAMtools (tested on v1.23)
ABRA2 (tested on v2.23)
Bamreadcount (tested on v1.0.1)
Java 8
Python (tested on v3.10)
brc‐parser.py (https://github.com/sridhar0605/brc‐parser)
Prokka (tested on v. 1.14.6)

1 Reference database construction: Index the reference genome database using BWA:


bwa index microbes.fa

  Here, microbes.fa is a FASTA file containing genome sequences of selected microbial taxa.
2 Read alignment to reference database: Align paired‐end sequencing reads to the reference database using BWA‐MEM:


bwa mem ‐t 16 microbes.fa read1.fastq read2.fastq > sample.sam

  This command maps paired‐end FASTQ reads (read1.fastq and read2.fastq) to the reference database using 16 threads. The output is a SAM file containing read alignments.
3 Conversion, sorting, and indexing of alignments: Convert the SAM file to BAM format, and then sort and index using SAMtools:


samtools view ‐@ 16 ‐b sample.sam > sample.bam
samtools sort ‐@ 16 sample.bam > sample.sorted.bam
samtools index sample.sorted.bam

  Optionally, remove intermediate files to conserve disk space:


rm sample.sam sample.bam

4 Local realignment of reads: Perform local realignment to improve detection of insertion and deletion events using ABRA2:


mkdir TMP
java ‐Xmx32G ‐jar abra2‐2.23.jar -in sample.sorted.bam -out sample.sorted.realign.bam -ref microbes.fa -threads 16 -tmpdir TMP -sa > sample.abra.log

  The “-sa” (skip assembly) flag reduces runtime by bypassing assembly‐based realignment.
  Optionally, remove intermediate folder:


rm ‐rf TMP

5 Retrieval of nucleotide coverage and variant information: Index the realigned BAM file:


samtools index sample.sorted.realign.bam

  Generate per‐base coverage and nucleotide variant information using *bam‐readcount*:


bam‐readcount ‐w1 ‐f microbes.fa sample.sorted.realign.bam > sample.brc.tsv

  This step produces a tab‐delimited file containing per‐base read counts and nucleotide composition across the reference genomes.
  Parse the output into a structured format using *brc‐parser.py*:


python brc‐parser.py sample.brc.tsv

  Convert the resulting CSV file to a tab‐delimited format for downstream analysis:


sed 's/,/\t/g' sample.brc_parsed.csv > sample_output.txt

**Figure 2:**
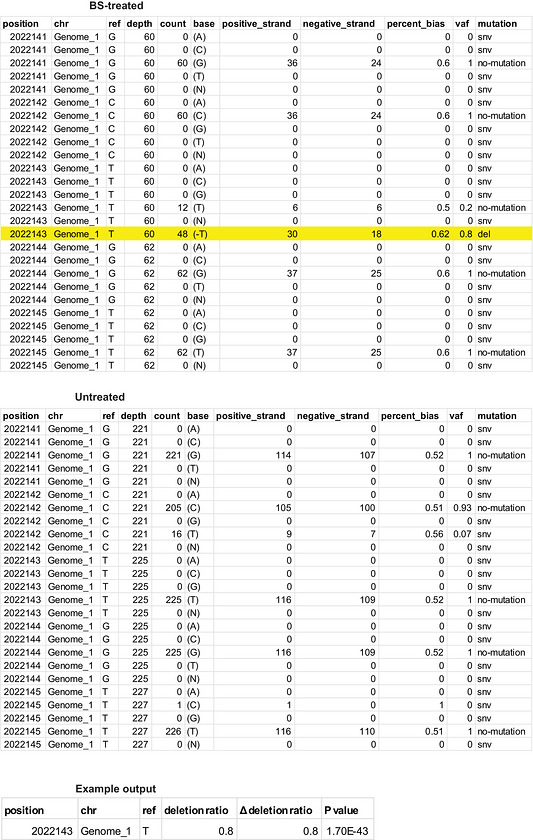
Representative output of pseudouridine detection pipeline in bisulfite‐treated and untreated microbiome samples. Tabulated output from the computational workflow showing per‐base nucleotide coverage, strand‐specific read counts, variant allele frequency (VAF), and mutation classification for selected genomic positions. In bisulfite‐treated samples (top), uridine positions exhibit characteristic deletion signatures (e.g., position 2022143, highlighted), represented as “del” events with substantial read support, consistent with bisulfite‐induced modification at Ψ sites. In contrast, the corresponding positions in untreated samples (middle) show little to no deletion signal, with reads predominantly matching the reference base (“no‐mutation”), indicating low background error rates. Columns include genomic position, reference base, total read depth, nucleotide counts, strand‐specific read distribution, percent strand bias, VAF, and mutation annotation. The final output table (bottom) summarizes positions that pass all filtering criteria for pseudouridine identification, including deletion ratio in bisulfite‐treated samples, Δ deletion ratio between treated and untreated samples, and statistical significance (*p*‐value).

  An example output file is shown in Figure 2.

6 Identification of pseudouridine sites: Uridine positions are classified as pseudouridylated if they meet all the following criteria:
  i. Read depth: ≥20 reads in both bisulfite‐treated and untreated samples
  ii. Deletion count (treated): ≥5 deletions in bisulfite‐treated samples
  iii. Deletion ratio (treated): ≥0.02 (2%) in bisulfite‐treated samples
  iv. Relative enrichment: Deletion ratio in bisulfite‐treated samples is at least twofold higher than in untreated samples
  v. Statistical significance: *p* < .01 by Fisher's exact test, comparing deletion counts and total read depth between treated and untreated samples
  For positions passing these thresholds, the level of pseudouridylation is estimated as the difference in deletion ratios between treated and untreated samples (Δ deletion ratio).
*IMPORTANT NOTE*: Deletions may also be observed in untreated samples because of strain heterogeneity in complex microbial communities. Therefore, stringent filtering criteria are required for accurate pseudouridine site identification as listed above. For single‐isolate bacterial analysis, the deletion ratio in untreated samples should be <0.01. In addition, the output file reports nucleotide coverage relative to the reference (sense) strand. For genes annotated on the antisense strand, adenine positions in the output correspond to uridine positions on the transcribed strand and should therefore be considered when identifying pseudouridylation sites.
  Strand annotation can be obtained from genome annotation files (e.g., GFF files from NCBI). Alternatively, gene prediction tools such as Prokka can be used to generate genome annotations, including strand information:


prokka -quiet -kingdom Bacteria -outdir out.dir -locustag genomes -prefix genomes microbes.fa

  An example test run can be found at https://github.com/EmiolaLab/bacPseudouridines.

## REAGENTS AND SOLUTIONS

### Bisulfite reagent (BSR)

Mix in 900 µl DEPC‐treated water (Thermo Fisher, AM9920):
  0.27 g sodium sulfite, anhydrous (Sigma Aldrich, 901916)
  0.034 g sodium bisulfite, anhydrous (Sigma Aldrich, 799394)

Store up to 1 day at room temperature. Always make fresh BS reagent for each library.

## COMMENTARY

### Background Information

A key feature of this protocol is its adaptation for microbiome‐derived RNA, which differs substantially from RNA obtained from single bacterial isolates or eukaryotic systems. Unlike conventional BID‐seq workflows developed for relatively homogeneous transcriptomes, microbiome samples contain mixed bacterial populations with highly variable transcript abundance, extensive rRNA content, and frequent strain‐level genomic variation. These characteristics introduce several technical challenges, including reduced mRNA representation, increased mapping ambiguity, and elevated background deletion frequencies in untreated samples. To address these issues, the present workflow incorporates optimized low‐biomass RNA extraction procedures, microbiome‐compatible rRNA depletion, stringent handling of fragmented RNA, and computational filtering strategies tailored for complex microbial communities. In particular, comparative analysis of BS‐treated and untreated samples, combined with conservative thresholds for deletion frequency and statistical significance, reduces false‐positive Ψ identification arising from strain heterogeneity and alignment artifacts. These microbiome‐specific modifications substantially improve the sensitivity and reliability of pseudouridine detection in complex bacterial communities.

### Critical Parameters and Troubleshooting

The most critical steps in this protocol are those that directly influence RNA yield and integrity. During the initial RNA extraction phase, inefficient cell lysis can result in suboptimal RNA recovery, whereas excessive heat generated during mechanical disruption may compromise RNA integrity. Therefore, lysis conditions must be carefully controlled, with intermittent cooling on ice between bead‐beating cycles to prevent heat‐induced RNA degradation while ensuring efficient cell disruption. RNA fragmentation represents another highly sensitive step, for which precise timing is crucial, as prolonged incubation or delayed addition of the stop reagent can lead to over‐fragmentation and degradation of RNA. Strict adherence to incubation times and immediate quenching are required to maintain optimal fragment size distribution. Additionally, after DNase treatment, particular caution must be exercised while collecting the RNA‐containing supernatant. The pellet formed during this step should remain undisturbed, as inadvertent disruption may introduce contaminants and negatively affect downstream applications.

A detailed troubleshooting guide summarizing common challenges and recommended solutions when carrying out these protocols is provided in Table 1.

**Table 1:** Troubleshooting Guide for Microbiome Pseudouridine Sequencing Workflow

Problem	Potential cause	Solution
Low RNA yield	Inefficient lysis	Increase the bead‐beating time on homogenizer.
No peak in final library check	Low RNA concentration	Final RNA concentration in 11 µl of sample must be in the range of 25‐500 ng; use Qubit HS RNA kit for estimating RNA yield.
	Poor RNA quality	RIN value of RNA must be ≥5.
	No index	Although the same anchors can be used in all samples, a unique index must be added in all samples of a library; increase the amount of index.
	RNA degradation due to over‐fragmentation	Optimize fragmentation by reducing the fragmentation time.
Peak at low fragment size (<190 bp)	Over‐fragmentation	Lower the fragmentation time by 1‐2 sec.

### Understanding Results

After sample processing and RNA extraction, the protocol is expected to yield RNA concentrations in the range of ∼7‐15 ng/µl, as measured by Qubit fluorometric quantification. RNA integrity is a critical determinant of downstream success and should meet a minimum RNA Integrity Number (RIN) threshold of 5. In validation experiments using oral microbiome samples, RIN values consistently exceeded 8 and, in some cases, reached 10; however, a RIN ≥5 is considered acceptable for subsequent applications. It is advisable to reassess both RNA concentration and integrity after the DNase treatment to confirm that these parameters remain within the required range.

Once the presence of RNA of sufficient quantity and quality has been verified, library preparation incorporating rRNA depletion can be initiated and carried through to completion (Fig. 1). The resulting libraries should be evaluated using the Agilent 2100 Bioanalyzer. Successful library construction is indicated by a single, well‐defined peak typically within the 200‐ to 500‐bp range.

Although fluorometric quantification provides a useful estimate of library concentration, it should not be used in isolation. High concentration readings may arise even in the absence of proper amplification or indexing, or due to the presence of adapter dimers. Therefore, fragment size analysis is essential to accurately assess library quality. After validation, libraries are subjected to sequencing, followed by computational analysis to generate the final outputs (Fig. 2).

The output of the computational pipeline provides per‐base nucleotide coverage, strand‐specific read counts, and variant annotations for each genomic position in both bisulfite‐treated and untreated samples. As shown in the example dataset (Fig. 2), most positions display high read depth with no detectable mutations (“no‐mutation”), indicating accurate alignment and low background error rates. At candidate Ψ sites, bisulfite‐treated samples exhibit characteristic deletion signatures at uridine positions, represented as “del” events (e.g., position 2022143 in Fig. 2), with a substantial fraction of reads supporting the deletion (variant allele frequency, VAF). This is also referred to as “deletion ratio.” In contrast, the corresponding positions in untreated samples show little to no deletion signal, confirming that these events are induced by bisulfite conversion rather than being sequencing or alignment artifacts. The comparison between treated and untreated datasets therefore enables discrimination of true Ψ sites. Positions that meet the defined filtering criteria (e.g., minimum coverage, deletion ratio, and statistical thresholds) are classified as pseudouridylated, and the extent of modification is quantified as the difference in deletion ratios (Δ deletion ratio) between treated and untreated samples.

A summary of the input requirements and expected performance is shown in Table 2.

**Table 2:** Recommended Quality Control Metrics and Sequencing Performance Benchmarks for Microbiome Pseudouridine Profiling

Metric	Recommendation/expected performance
Expected RNA yield	7‐15 ng/µl
RIN requirement	≥5
rRNA depletion performance	Variable by sample type and RNA quality; typically ∼5‐30% residual rRNA in microbiome samples.
Library size	200‐500 bp
Sequencing depth	>30 million paired‐end (PE) reads pdf sample; 50‐100 million PE reads often preferred for complex metatranscriptomes.
Mapping rate	Highly variable—depends strongly on host contamination, microbiome complexity, and reference database completeness.

### Time Considerations

Collection of microbiome samples can be time‐intensive; however, specimens preserved in RNA stabilization buffer may be stored directly at −80°C for extended periods without compromising RNA integrity, provided that repeated freeze‐thaw cycles are avoided. Initial sample processing, including RNA extraction, DNase treatment, and quality assessment via fluorometric and electrophoretic methods, typically requires 5‐6 hr, depending on sample throughput. After quantification, RNA samples may be stored at −80°C prior to library preparation.

Subsequent steps, including mRNA enrichment, bisulfite treatment, and cDNA synthesis, must be performed consecutively without interruption and generally require 10‐12 hr to complete. Pauses or overnight storage during this phase are not recommended, as they may adversely affect RNA integrity and reaction efficiency. Final library preparation steps, including indexing, multiplexing, and quality control, require an additional 4‐5 hr. Completed libraries are stable for up to 1 month at −20°C or for extended durations at −80°C, provided freeze‐thaw cycles are minimized. Overall, the complete protocol spans ∼3‐4 days, depending on sample number and workflow organization.

### Author Contributions

**Shikha Sharma**: Conceptualization; methodology; writing—review and editing. **Ning Duan**: Methodology; visualization. **Akintunde Emiola**: Conceptualization; methodology; writing—review and editing.

### Conflict of Interest

The authors declare no conflicts of interest.

## Data Availability

The data that support the findings of this study are available from the corresponding author upon reasonable request. Codes are publicly available in the Github repository (https://github.com/EmiolaLab/bacPseudouridines).
